# Dynamic response of thickened tailings in shaking table testing

**DOI:** 10.1186/s40703-021-00156-1

**Published:** 2021-10-04

**Authors:** Fahad Alshawmar, Mamadou Fall

**Affiliations:** Department of Civil Engineering, University of Ottawa, 161 Colonel by, Ottawa, ON K1N 6N5 Canada

**Keywords:** Tailings, Mine, Dynamic loadings, Tailings dam, Thickened tailings

## Abstract

In this study, an instrumented thickened tailings deposit model was designed, built and employed to evaluate the behaviour of layered thickened tailings to dynamic loading by using a shaking table equipment. The thickened tailings were deposited subsequently in three thin layers in a flexible laminar shear box mounted on top of the shaking table. Cyclic loading with a peak horizontal acceleration of 0.13 g and a frequency of 1 Hz was applied to the layered tailings deposit. Different types of sensors were placed to monitor the accelerations, displacements, volumetric water content and pore water pressures at the intermediate depth of each layer. Results indicated that the acceleration for the bottom and middle layers were similar to that of the base of the shaking table; but, this was not the case for the top layer. The measurements of vertical displacements indicated that all layers of thickened tailings experienced initially contraction and subsequently dilation during the shaking. The excess pore water pressure ratios were found to exceed unity through all layers of thickened tailings when the shaking ended. In other words, the results showed that the layered thickened tailings are susceptible to liquefaction under the considered testing conditions. It is also found that upward pore water migration to the top layer and downward pore water flow to the bottom layer occurred in the thickened tailings deposit. This water migration generated additional pore water pressure and also impacted the vertical displacement and liquefaction susceptibility of the thickened tailings material. The results of this study give a better understanding of the dynamic behaviour of thickened tailings, which is crucial for the safety of thickened tailings systems as well as sustainable mining.

## Introduction

Mining operations globally generate large quantities of tailings materials every year [58]. Jones and Boger [44] reported that the mining operations generated 51 billion tons of waste rock and 14 billion tons of tailings in 2010. Tailings materials are considered ground rock particles in which the valuable minerals are recovered [9, 29, 30, 33, 48, 50, 64], where the size of tailings is generally ranged from silt to sand [32, 34]. Tailings are deposited conventionally as a slurry in a structure called tailings dam [1, 24, 25, 33, 60, 90, 92]. The deposited slurry tailings in the dam are considered typically soft, loose and constantly saturated [43, 55]. Consequently, they are susceptible to liquefaction phenomenon when subjected to any strong earthquake shaking or seismic loadings [36]. Tailings dam failures induced by earthquakes have been reported since the early 1900’s (e.g., [15, 45]. In the event of a liquefaction, the tailings may be released through a breach in the retaining embankment. The result is comparable to a landslide, with the consequences frequently being the fast release of large quantities of toxic slurry tailings.

The term of liquefaction phenomena has been defined as “a condition where a soil will undergo continued deformation at a constant low residual stress or with no residual resistance, due to the buildup and maintenance of high pore-water pressures which reduce the effective confining pressure to very low value” [59]. Even though the historical events have indicated that the mine tailings dams have experienced more static liquefaction than seismic liquefaction, the liquefaction phenomenon is mostly related to the cyclic loading conditions [18]. The liquefaction due to earthquake events is considered the second cause of tailings dam failures around the world whilst it is not the case in Europe [66]. Severe consequences such as the loss of life, injuries, and environmental damage from the previous tailings dam failures, due to the liquefaction during the earthquake, have been reported in the literature (e.g. [8, 34, 38, 86, 89]). The attention of the society, scholars, and government has been attracted as a result of such seismic failure. Thus, dewatering the slurry tailings before the deposition on the surface is considered an appropriate approach/method to prevent or diminish such consequences that might be resulted from the failures of tailings dams induced by liquefaction. Thickened tailings (TTs) technology is one of the novel dewatering techniques that can be used to significantly dewater low solids concentrated slurry tailings. This is usually accomplished by means of compression thickeners or by combined thickeners and filter presses.

The thickened tailings (TTs) are defined as tailings from which a considerable amount of process water has been removed to reach solids contents of higher than 50% and discharged out through spigot [17, 22, 67]. In addition, such thickened tailings material usually displays non-Newtonian flow behaviour [77]. Robinsky [69] is considered to be the pioneer of this technology which was initially implemented at the facility of the Kidd Creek Mine in Ontario, Canada. TTs have more solids content by weight compared with the slurry tailings as well as can be pumped as in the case of slurry tailings [17]. The solid content in thickened tailings is ranged between 50 and 70% with a yield stress of between 30 and 100 Pa [31, 76]. Unlike slurry tailings during the deposition, the segregation of thickened tailings does not occur since the mass of thickened tailings has a homogenous behaviour with low permeability [67]. The high-density thickened tailings will flow up to stop generally at slope beaches ranging from 2 to 10% [23]. Thickened tailings disposal method has several advantages. It enables to (i) reduce or eliminate the risk and consequences of tailings dam failure, (ii) increase the amount of water available for reuse, (iii) decrease the footprint of the tailings storage facility, (iv) provide an option for progressive closure, and (iv) reduce the leachate seepage risk [16, 22, 67, 69].

There have been numerous available laboratory studies on the seismic or cyclic behaviour of tailings in the literature which were carried out by different researchers [3, 4, 14, 16, 28, 39, 41, 74, 88]. However, most of these studies were conducted on conventional slurry tailings. Moreover, almost all the past laboratory seismic or cyclic studies were based on either cyclic direct simple shear or triaxial apparatuses. Yet, to the best knowledge of the authors, there is only one study that used shaking table testing technique to investigate the dynamic behaviour of tailings. Indeed, [61, 62] have investigated the cyclic behaviour of “conventional” or slurry tailings (hard rock tailings) by using shaking table equipment. However, the results of this study cannot be applied to TTs because TTs are different than “conventional” tailings. However, no studies have been conducted on the cyclic behaviour of layered thickened tailings (TTs) by using shaking table testing technique. The liquefaction susceptibility of TTs is not well-known. An understanding and evaluation of the cyclic behaviour and liquefaction susceptibility of TTs is required before the use or implementation of this TT deposition technique in earthquake-prone areas. Regulators are concerned with potential liquefaction of TTs deposits in those regions. There is a need to address this research gap.

The main objective of this study is to evaluate the behaviour of layered thickened tailings under dynamic (cyclic) loading conditions using a shaking table. Three different layers of thickened tailings were deposited in the flexible laminar shear box (FLSB). This paper presents and discuss the experimental results including the acceleration and horizontal displacement, pore water pressure, vertical displacement/settlement, and liquefaction responses.

## Materials and equipment used in the experiments

### Materials

The thickened tailings (TTs) were created from mixing natural tailings (NTs) and industrial silica tailings (STs) with municipal water. The usage of these types of tailings together as a mixture allowed the accurate control of the mineralogical and chemical compositions of the tailings materials, thereby reducing uncertainties in the results obtained to a minimal level. Indeed, natural tailings can contain numerous reactive minerals, such as sulphide minerals. The latter, when exposed to air during the preparation and/or deposition of the tailings, can be oxidized. This oxidation can change the initial chemical or mineralogical composition of the TTs and/or result in the precipitation of reaction products, which may cement some tailings particles together.

**Fig. 1:**
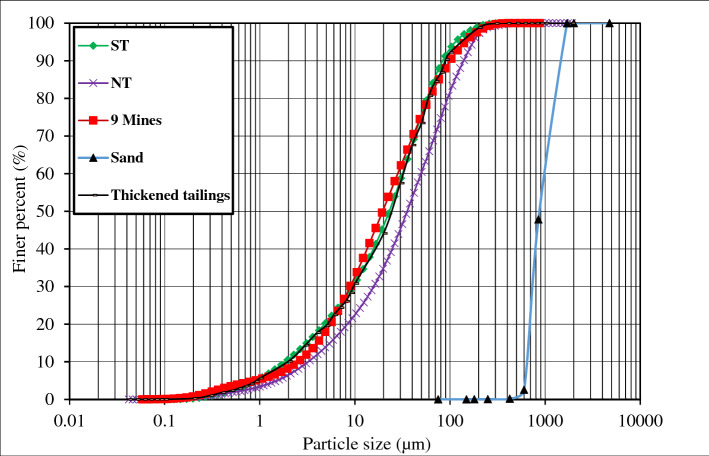
Particle size distribution of the tailings and coarse-grained sand used in this work compared with the average of 9 eastern Canadian mine tailings

NTs were sampled from a mine that is located in eastern Canada. STs contain 99.8 wt% silicon dioxide (SiO_2_). The key physical properties and mineral compositions of the used tailings (NTs; STs) are listed in Tables 1 and 2. NTs, STs, and their mixture show a particle size distribution (Fig. 1) close to the average of 9 mine tailings that are coming from eastern Canada [56, 57, 63]. From this figure, it can be said that NTs and STs include almost 37% and 45% particles finer than 20 µm, respectively. Based on the classification of tailings, these used tailings are considered as medium tailings [46]. Tests to determine the liquid and plastic limits of the used tailings were carried out following the experimental procedure as in the ASTM D4318-17e1 [5]. Based on the laboratory work, the used tailings were non-plastic and could not be rolled (plasticity index, PI ~ 0). According to the Unified Soil Classification System (USCS), the used tailings are categorized as sandy silts of low plasticity (ML). Therefore, under the conditions of the seismic loading, the anticipation for this mixture of tailings (i.e. TTs) to be subject to liquefaction phenomena can be considered very likely with the absence of plasticity in the tailings [61].

**Table 1:** Physical properties of the tailings used and the coarse-grained sand

Material	Physical properties						
	G_s_	D_10_ (µm)	D_30_ (µm)	D_50_ (µm)	D_60_ (µm)	C_u_	C_c_
STs	2.7	1.9	9.0	22.5	31.5	–	–
NTs	3.1	3.2	15.8	35.5	49.5	–	–
Sand	2.7	700.0	740.0	880.0	1000.0	1.4	0.8

*STs* silica tailings, *NTs* natural tailings, *G*_*s*_ specific gravity, *C*_*u*_ coefficient of uniformity, *C*_*c*_ coefficient of curvature

**Table 2:** Mineralogical composition of the tailings used

Tailings	Mineral											
	Quartz	Albite	Dolomite	Calcite	Chlorite	Magnetite	Pyrite	Talc	Pyrrhotite	Spinel	Others	Total
STs (wt.%)	99.8	–	–	–	–	–	–	–	–	–	0.2	100.0
NTs (wt.%)	15.0	32.8	15.0	4.2	16.1	2.4	1.0	7.0	1.8	1.8	2.9	100.0

*STs* silica tailings, *NTs* natural tailings

### Preparation of the thickened tailings

The mixture of 10% of NTs and 90% of STs with sufficient municipal water was prepared to reach the consistency of the thickened tailings. In the preparation stage, a large concrete mixer was used to mix the thickened tailings material until reaching homogeneity or obtaining a homogeneous material. The mixing time for all mixes was lasted for about 10 min. Wykeham-Ferrance laboratory vane shear apparatus was used for the workability evaluation and the yield stress measurement of this prepared thickened tailings material. From a single-point measurement (the maximum torque), the yield stress of the prepared thickened tailings can be determined [20]. It was found that the yield stress was about 63 Pa which is considered related to the characteristic of the thickened tailings as stated, for example, by Sofra [76]. This amount of yield stress was equivalent to a solid content of about 70%. The degree of saturation of the prepared thickened tailings was determined to be equal to 100% (the degree of saturation, S, was calculated according the equation, S = (w.G_s_/e; w: water content; G_s_: specific gravity; e: void ratio).

### Shaking table test

The behaviour of soils under seismic or cyclic loading conditions has been often investigated through the physical modeling tests (i.e. 1-g shaking table or centrifuge testes). These physical modeling tests have certain advantages and limitations [21, 72]. The well-controlled large amplitude, easier experimental measurements, and understanding the basic mechanisms of failure are considered advantages of the shaking table tests as compared with the centrifuge tests. However, the centrifuge tests can be used for measurements under field stress conditions which is not the case for the shaking table tests. Moreover, there are two issues, which associated with the centrifuge tests, that include all laws of scaling can not be fulfilled instantaneously and the response of whole soil can not be measured through a dense sensor set.

**Fig. 2:**
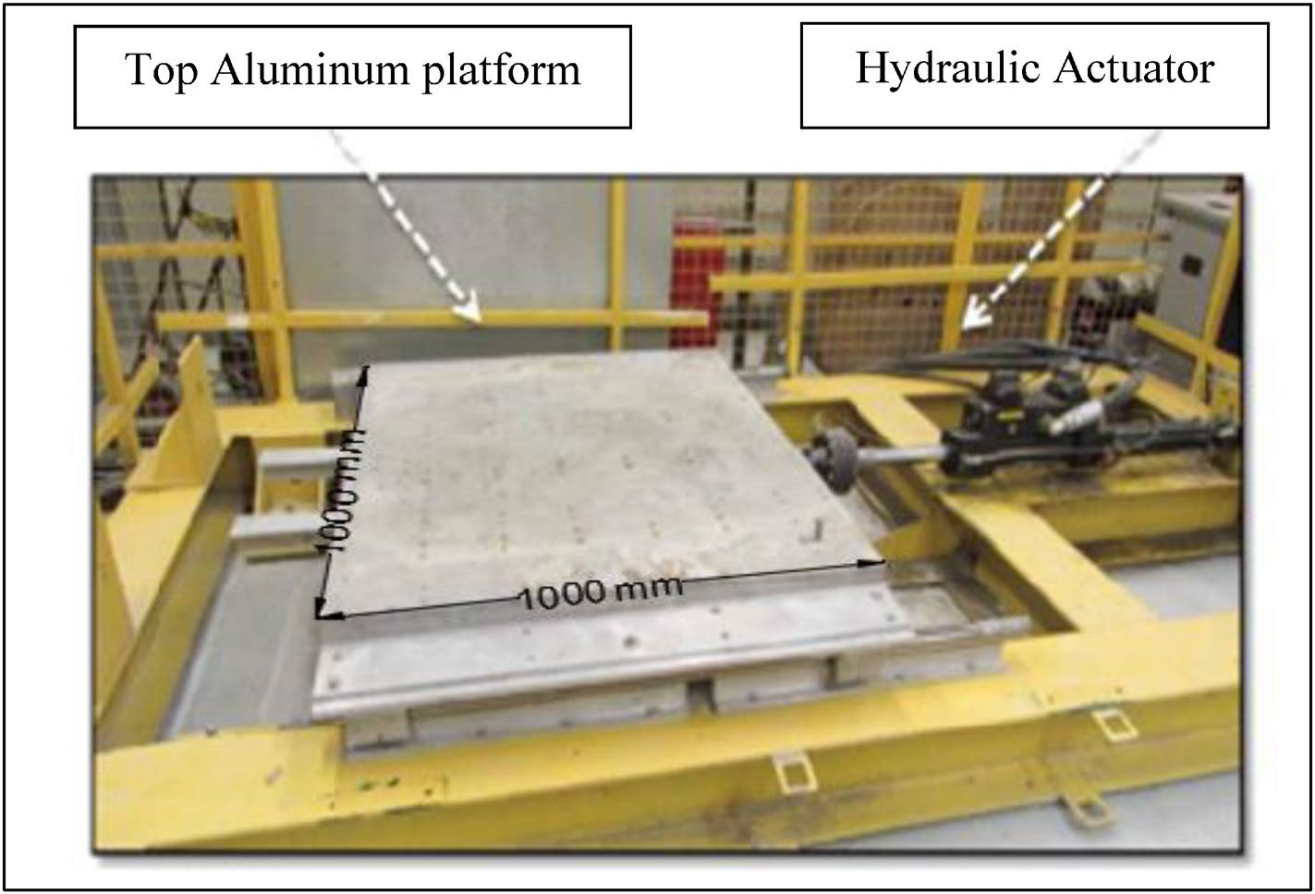
Shaking table equipment at the University of Ottawa used for this research (photo from [7]

In this paper, the shaking table equipment was used to simulate the cyclic behaviour of the thickened tailings under the one direction of shaking loading. This used shaking table equipment is illustrated in Fig. 2. As shown in the figure, this equipment has dimensions of 1000 mm × 1000 mm as a top aluminum platform capable of carrying a maximum payload of 1000 kg. A hydraulic actuator (model 244) with its capacity of 25 kN (Fig. 2) can move the shaking table horizontally up to a frequency of 17 Hz with a maximum allowable peak to peak displacement in the longitudinal direction is 0.12 m.

### Model construction, setup and instrumentation

A flexible laminar shear box (FLSB) is adopted to be used in this research work instead of a rigid box since the flexible laminar shear box (FLSB) permits the shear deformation of the tested specimen while providing enough confinement [12]. Therefore, this leads to more simulation of the free-field boundary conditions when compared with the rigid box.

**Fig. 3:**
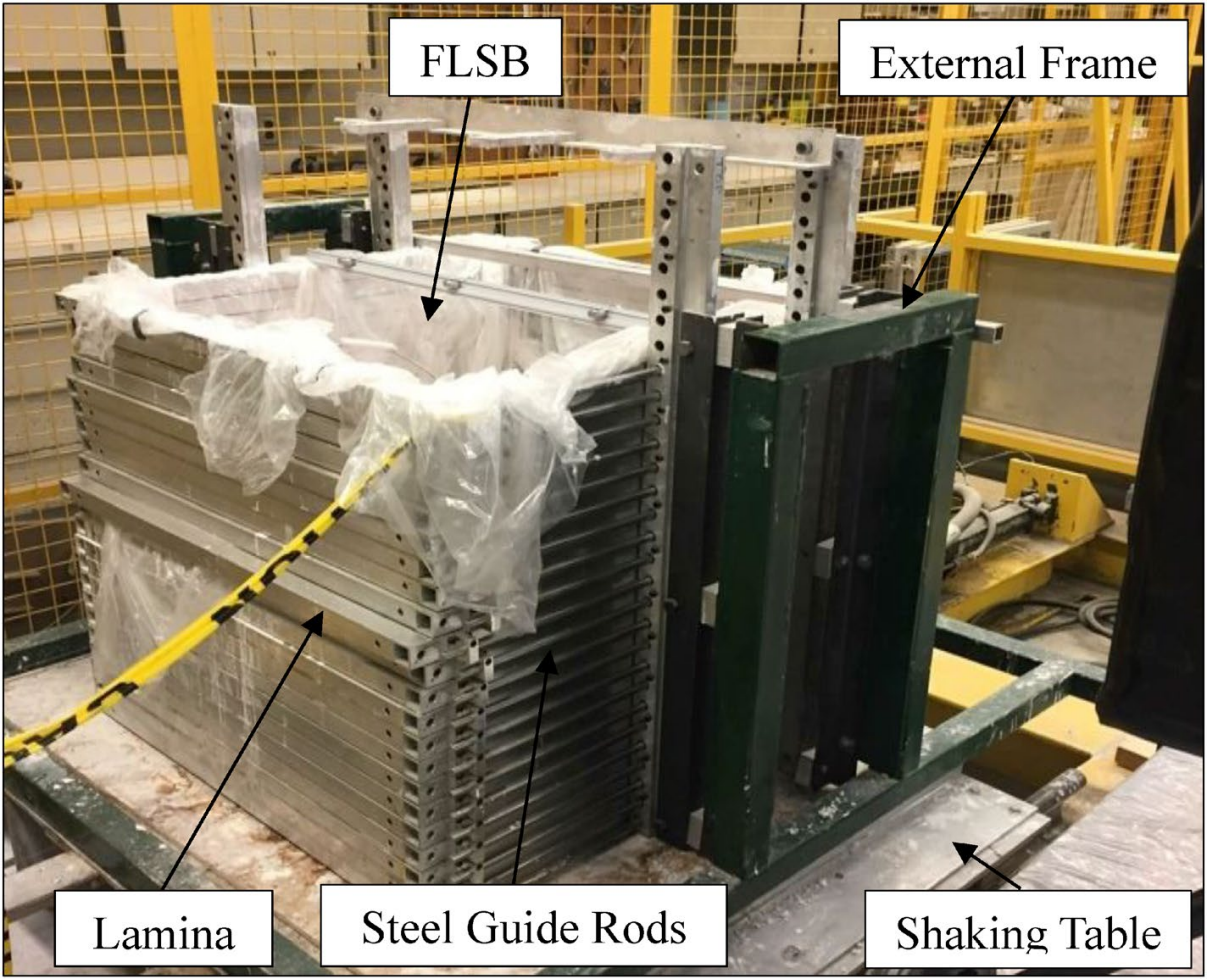
Snapshot of the University of Ottawa FLSB (designed by [2]

The FLSB was made-up to be suitable for the shaking table equipment at the civil engineering laboratory at the University of Ottawa. Designing of this FLSB was performed by Alainachi and Fall [2] while it was constructed in the machine shop at the University of Ottawa. In addition, the design of the used flexible laminar shear box (FLSB) herein is considered to be like the design of those used by other researchers [53, 80]. A snapshot of the FLSB is given in Fig. 3. The volume of this FLSB is about ~ 0.422 m^3^ (i.e., dimensions: 750 mm in length, 750 mm in width, and 750 mm in height) which was formed by staking a set of 22 horizontal laminae on top of each other with a small distance (i.e. 2 mm) between each one of them to permit for the relative movement. Aluminum material was used to construct these laminae (i.e. box Sects. 31.65 mm × 31.65 mm) since it has somewhat lightweight and, at the same time, can provide enough confinement. The laminae were individually supported through linear bearings and stainless-steel guide roads in which connected to an external frame. These stainless-steel guide roads (two with 15 mm as diameter) were connected to the exterior wall of each lamina by stiff aluminum brackets. Six ball bearings with low friction, per lamina, were installed to feed these guide roads through them in which were fixed in four vertical bearings brackets. The vertical bearings brackets were then connected to the external frame. In general, the used external frame herein is considered to be like those available in the literature [53, 80]. The effect of inertia that can occur due to the mass of the empty FLSB (~ 132 kg) can be removed by transferring the total mass to the external frame. The mass on the external frame is carried then by big steel beams and consequently to the ground of the test site.

**Fig. 4:**
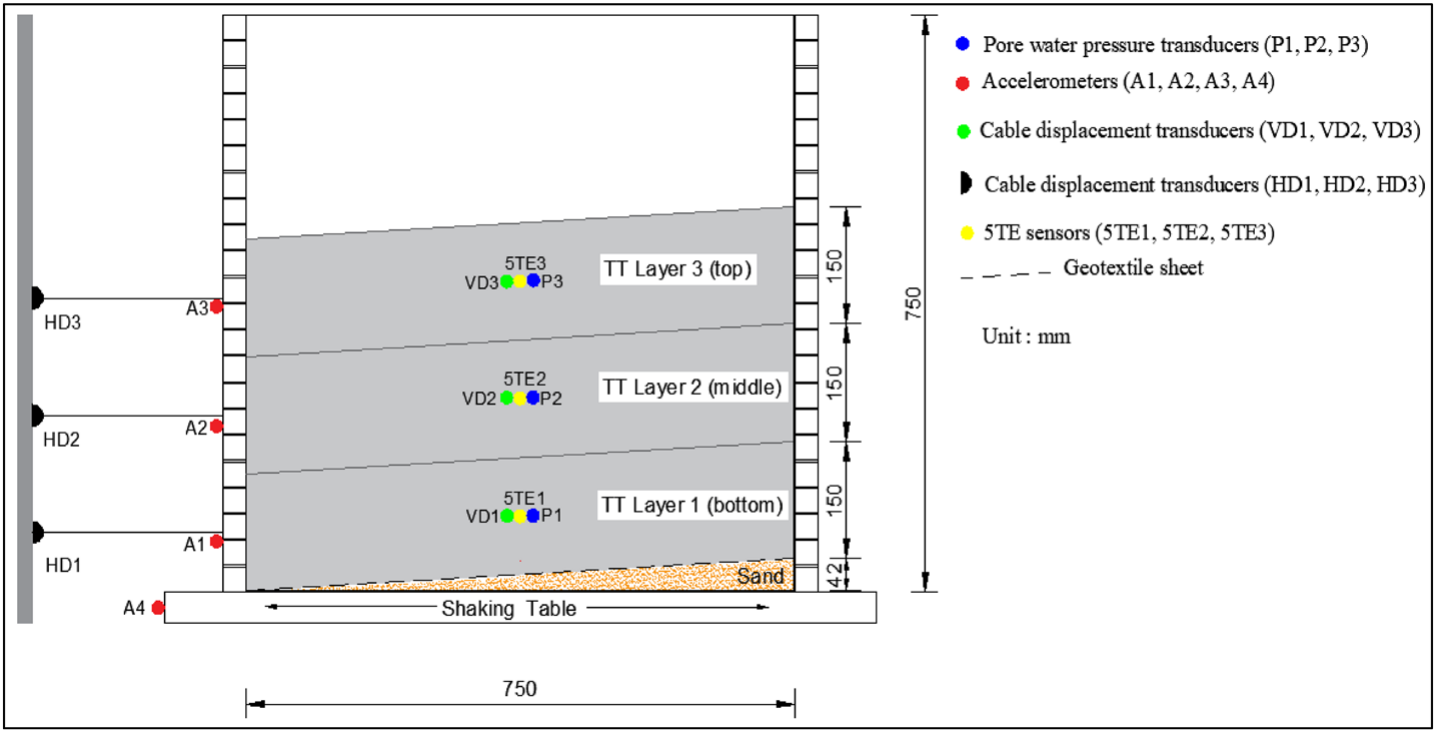
The used Flexible Laminar Shear Box (FLSB) on the shaking table with the positions of sensors

In thickened tailings technology, the tailings are subject to the dewatering process, where then they flow away from single or different deposition points to form gently sloped self-supporting stacks [52]. To simulate such slopes in this experimental work, a layer of sand material was, used underneath the thickened tailings deposit, placed inside the FLSB to form a slope with an angle of approximately 5.6% as shown in Fig. 4. This selected slope angle herein was within the range that is reported in the literature as well as applied in the mining industry [23, 68]. It should be underlined that, in practice, the slope angle in the field is a function of several factors, such as reclamation and climatic conditions, solid content, grain size distribution and pH of the TT, and the rate of discharge per spigot [68]. The used sand is categorized as a poorly graded sand (SP) based on the Unified Soil Classification System (USCS). Table 1 illustrates the physical characteristics of this sand layer while Fig. 1 presents the particle size distribution curve for this sand layer.

A number of sensors were used to monitor the behaviour of the thickened tailings deposit during and after the shaking test. Figure 4 illustrates the positions of the sensors along with the FLSB. Three accelerometers (numbered as A1 to A3) were used for monitoring the acceleration behaviour of the tailings (i.e. at the intermediate depth of each layer) and were placed along the left external wall of the FLSB, on the same direction of shaking. To measure the motion of shaking, one accelerometer (numbered as A4) was also placed on the shaking table. These accelerometers (type 7593A) are from Endevco Company and have an acceleration range of ± 2 g. Three cable displacement transducers (numbered as HD1 to HD3) were used for monitoring the horizontal displacement behaviour of the tailings (i.e., at the intermediate depth of each layer) which were installed on a steel pole and then stretched to the left external wall of the lamina on the same direction of shaking. Additional three cable displacement transducers (numbered as VD1 to VD3) were used for monitoring the settlement of the tailings (i.e. at the intermediate depth of each layer) which were hanged on a steel bridge that was attached on the top middle of the FLSB. These cable displacement transducers (numbered as VD1 to VD3) were then tied on the upper section of steel rods while the lower section of these steel rods was then connected to perforated plastic plates which their settlement lead to pull the cables down. Mesh cylinders (made from light steel) with open holes were used to keep the steel rods from falling during the shaking. Such a way was used previously by Alainachi and Fall [2]. All cable displacement transducers (type SM2-12) are manufactured by Measurement Specialties Company and have a range of ± 318 mm. Other sensors were embedded in the tailings deposit (i.e. at the intermediate depth of each layer) on the normal direction of shaking which include three pressure transducers (numbered as P1 to P3) for pore water pressures monitoring and three 5TE sensors (numbered as 5TE1 to 5TE3) for volumetric water content monitoring. These pressure transducers (type PX309-015CG5V series) are manufactured by the Omega Company and have a pressure range of ± 103.42 kPa. The 5TE sensors are manufactured by Decagon Devices Inc. and monitor the volumetric water content within the range of 0–80%. These embedded sensors and their cables are in small size, water resistance, and durable.

### Thickened tailings deposition, testing program and procedure

The deposition process of the thickened tailings in the FLSB was performed in three thin layers (i.e. each layer had a thickness of 150 mm) to simulate the field deposition or field layer thicknesses as in the in-situ [65], as shown in Fig. 4. These layers were named in the order according to their deposition in the FLSB (Fig. 4). The deposition time between the first layer (bottom layer) and the second layer (middle layer) was three days while it was two days between the second layer (middle layer) and the third layer (top layer). Deposition of thin layers and desiccation (drying) are key in order to increase the strength of the tailings prior to pouring the next fresh layer [47]. Although thin-layer deposition method and desiccation promote strength development, which, in turn, enhances the mechanical stability of the thickened tailings structure, an excessive drying could lead to the oxidation of sulphide minerals (e.g., pyrite) in sulphidic tailings. This oxidation can result in the generation of acid mine drainage, thereby jeopardizing the environmental safety of the thickened tailings disposal technique.

**Fig. 5:**
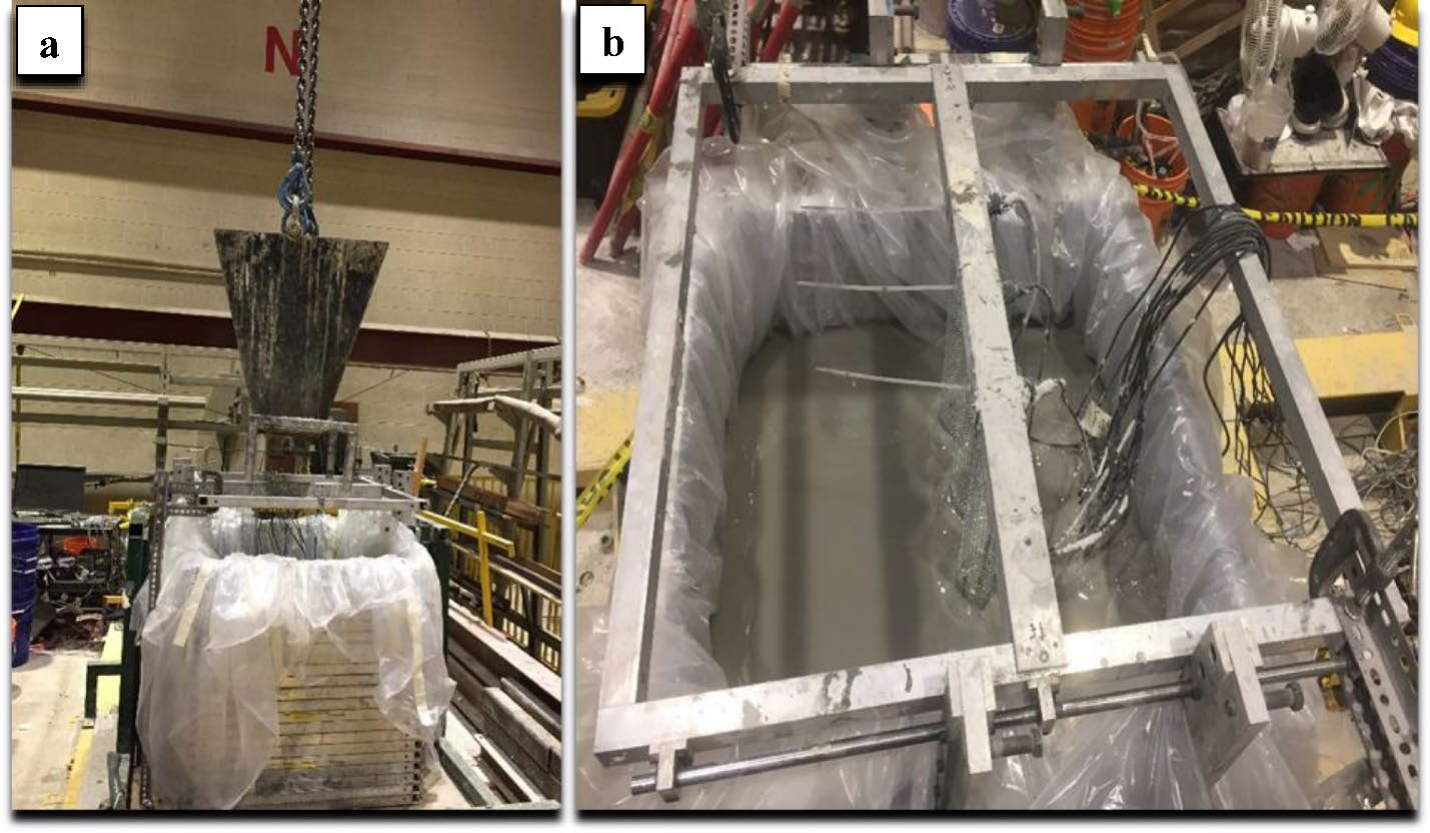
**a** Snapshot showing the concrete bucket that was used for deposition of thickened tailings layers. **b** Snapshot of the ground surface of thickened tailings shortly after deposition of the third (top) layer

In the deposition of the aforementioned thin layers, a concrete bucket that hanged by a crane was used. This concrete bucket has an opening at its bottom for pouring the thickened tailings in the FLSB as shown in Fig. 5a. The surface of the fresh thickened tailings deposit can be shown in Fig. 5b. A temperature and humidity sensor was used in the laboratory and showed that the average temperature and relative humidity were 20.4 °C and 57%, respectively.

The experimental program of this study comprised from testing of the thickened tailings deposit at early deposition by using the shaking table. It should be pointed out that the goal of this study is to evaluate the dynamic or cyclic behaviour of thickened tailings at early ages, i.e. when the deposition of the first layers of thickened tailings has been completed. This approach enables to appropriately scale down the parameters of the model experiment based on the similitude laws. Thus, a thickened tailings structure after the deposition of three 150 mm-thin layers of thickened tailings was considered in this testing program. In practice, this will correspond to the early ages (first week) of the deposition of thickened tailings. Though only three layers of thickened tailings or the thickened tailings during early deposition were tested in this research, gaining insight into the response of 3 layers of thickened tailings or thickened tailings at early ages to dynamic loading will permit to gain a deeper understanding of the cyclic behaviour of thickened tailings deposits at advanced or mature ages, i.e. when the deposit is made of over 30 layers. Moreover, considering a thickened tailings deposit with 3 layers enabled to meet the key similitude requirements between the physical model and prototype. Hence, a geometric scaling factor (N) of 1:1 was adopted for the experimental shaking table tests on the scale model in this study. The geometry and configuration of the models were designed based on the similitude theories associated with the 1 g shaking table test for the soil-fluid models proposed by Iai [35] and Meymand [51].

The dynamic (cyclic) loading conditions adopted in this study are similar to those applied in previous studies on cyclic behaviour of slurry tailings (e.g., [42, 61, 62]. The testing program includes applying a peak acceleration of 0.13 g and a frequency of 1 Hz at the bottom of the thickened tailings deposit that contained inside the FLSB for a time of 1800s (30 min). The minimum level of the generated peak accelerations, due to seismic earthquakes shakings, that needed to cause the liquefaction phenomena were reported in the literature to be 0.05–0.10 g [10]. Accordingly, the mine tailings in several regions in eastern Canada might be susceptible to the liquefaction phenomena under the generated peak accelerations as a result of the seismic earthquakes shakings [42]. The amount of acceleration in this investigation was similar to the acceleration that resulted during the earthquake that occurred in Saguenay, Québec, Canada, which had a scale of 5.9 [81]. It should be emphasized that only the peak acceleration is within the range of acceleration produced by Saguenay Earthquake, not the whole time series. The selected applied frequency herein was based on previous liquefaction studies (e.g. [28, 39, 42, 61, 62, 84]. and compatible with the capability of the used sensors in terms of the monitoring. The instruments used were unable to monitor the dynamic response of the tested thickened tailings or soils under large loading frequencies. Even though the duration of shaking in the actual earthquakes is much shorter than that one used in this study, such a long duration of shaking (e.g. 1800s) will permit the insight into the cyclic behaviour of the tested tailings as well relative comparisons of their behaviour. This is considered vital in terms of the future development of a constitutive model in order to expect and evaluate the dynamic behaviour of the thickened tailings. Previous authors have used a similar long duration of seismic shaking in their liquefaction studies of hard rock tailings (e.g. [42, 61]. It should be emphasized that these selected dynamic loading conditions were not intended to simulate a natural earthquake. Unlike natural earthquakes, the signal used in this study was one-dimensional, of uniform amplitude, constant frequency, and of long duration. In natural earthquakes, the number of cycles is a function of the earthquake magnitude (and is commonly of the order of 5 to 20), and the motion amplitudes have a gradual increase, and a gradual decrease. However, the dynamic loading conditions selected in this study enabled to gain a deeper insight into the cyclic behavior of this type of tailings as well as provide key information necessary for the future development of model to describe the seismic behaviour of tailings. Comparable types of loading have often been used in other cyclic behaviour and/or liquefaction studies (e.g., [19, 26, 27, 40, 42, 61, 62]. The bottom of the thickened tailings deposit was subject to shaking after 24 h from the deposition of the top layer. The behaviour of the tested tailings was continued to be observed once the shaking was stopped. Once the excess pore water pressure experienced a completed dissipation condition, the measurements through the sensors were stopped.

Several procedures were followed prior to the deposition of the thickened tailings deposit in the FLSB. The interior of the FLSB was first covered by a thin flexible plastic bag to pour the materials inside it, as shown in Fig. 3. This action was done to inhibit the leakage of the poured materials and water. Then, a drainage layer of sand material, with a slope of 5.6%, was poured on top of the plastic bag. A geotextile material was then placed between the sand layer and the tested tailings. This allowed only the water to move from the tailings materials layer to the sand layer without passing the particles of tailings. Finally, the three thin layers of the thickened tailings were deposited sequentially, as described above, in the FLSB with a total height of 450 mm (Fig. 4).

## Results and discussions

### Acceleration and horizontal displacement response

**Fig. 6:**
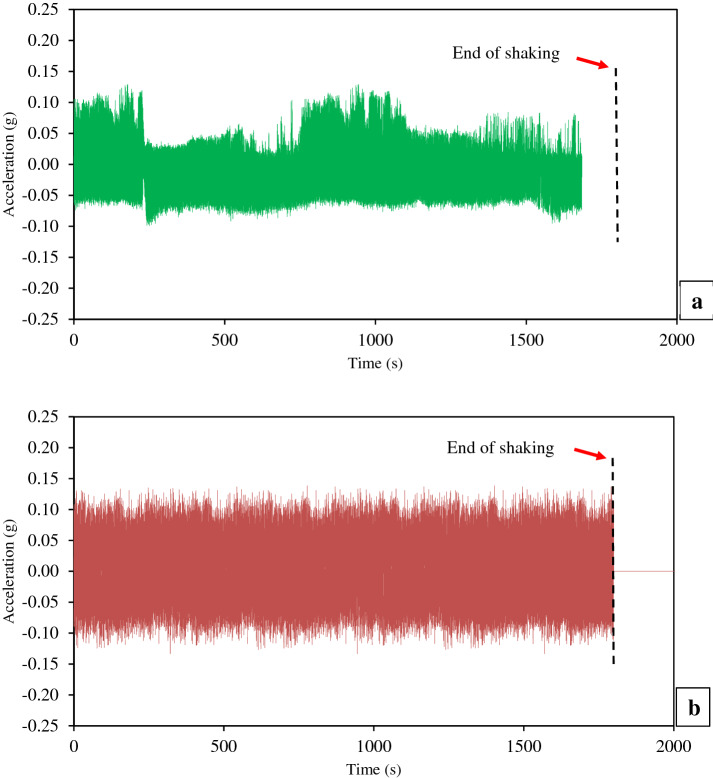

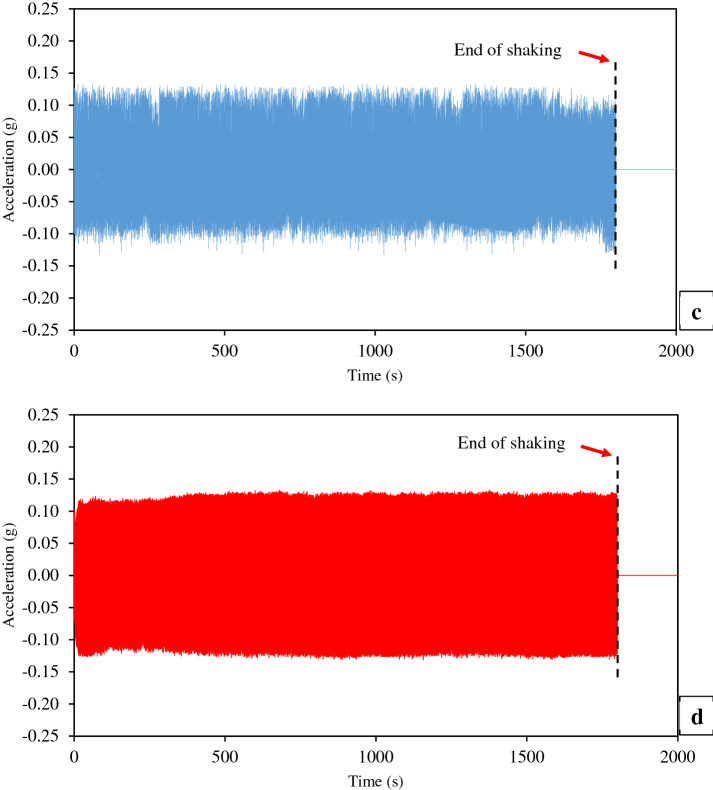
Time histories of measured accelerations at the intermediate depth of each layer of thickened tailings deposit and at the base of the shaking table during shaking: **a** Layer 3 (top), **b** Layer 2 (middle), **c** Layer 1 (bottom), **d** shaking table

**Fig. 7:**
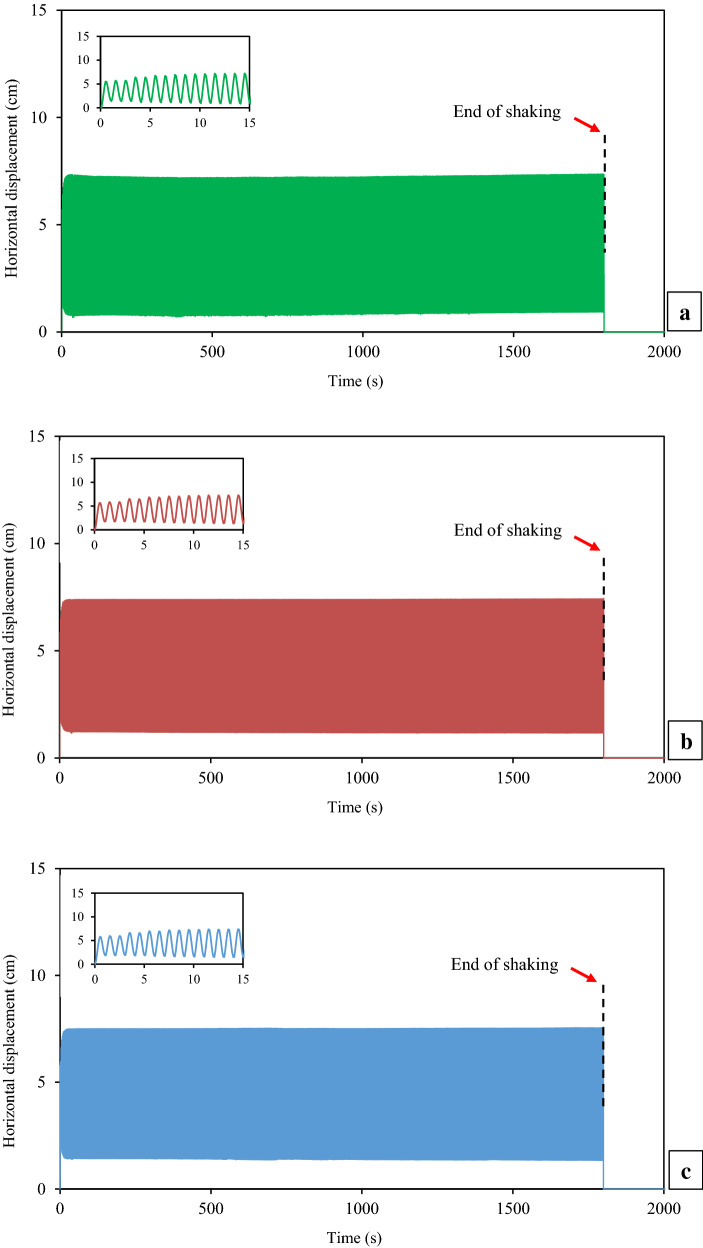
Time histories of measured horizontal displacements at the intermediate depth of each layer of thickened tailings deposit during shaking: **a** Layer 3 (top), **b** Layer 2 (middle), **c** Layer 1 (bottom)

The acceleration response is considered as one of the ways that can be used as an indication of the liquefaction occurrence [6, 78]. For instance, Ueng et al. [83, 84] were reported the use of the measured accelerations at different depths of sand as a way for the determination of the liquefaction occurrence. Figure 6 reveals the acceleration time histories of measured accelerations at the shaking table and intermediate depth of all layers of the thickened tailings. Similarly, Fig. 7 shows the horizontal displacement time histories of measured horizontal displacements at the intermediate depth of all layers of the thickened tailings. As depicted in these figures, the termination of shaking loading can be pointed out by the red arrows.

As expected, the measured acceleration at the base of the shaking table increased rapidly and attained almost the sinusoidal input acceleration (0.13 g), as shown in Fig. 6d. This indicated that the system of the shaking table was working well. The results revealed that the measured accelerations, at the intermediate depth of the bottom and middle layers, were seen to be not diminution and somewhat similar to the trend of the measured acceleration at the base of the shaking table (no phase shift was observed in the comparison of the cycles). This means that the applied sinusoidal input acceleration propagated entirely to these two layers. Coelho et al. [13], who carried out cyclic loading tests on saturated sand using centrifuge apparatus, observed similar behaviour. They stated that the reason for the aforementioned behaviour might be related to the large value of the initial vertical stress at these two layers. However, this Coelho et al.’s explanation may not be valid for study because of the low vertical effective stress. Future studies should confirm this explanation or clarify the cause of this behaviour. On the other hand, the measured acceleration at the intermediate depth of the top layer found to be not regular and diminution relative to the measured acceleration at the base of the shaking table, as revealed in Fig. 6a, d. Such behaviour could be resulted from the occurrence of the liquefaction where this top layer of thickened tailings was experienced nonlinearity, strength and stiffness reduction as a result of the applied shaking loading. However, it should be mentioned that other or additional factors could have caused the observed changes of acceleration in the top layer. These factors could be higher effect of excess pore pressure and reduction in shear resistance as well as increase in damping ratio.

As shown in Fig. 7, the measured horizontal displacements at the intermediate depth of all layers of the thickened tailings were noticed to be increased rapidly and almost similar to each other. Throughout the shaking loading, this behaviour is compatible with the observation of the motion of FLSB by the naked eye. However, the detailed observation of the recorded data shows that during shaking, the upper layer has slightly larger horizontal displacement than the lower or bottom layers. For example, the measured horizontal displacement in the top layer after 4.5 s of shaking is 10% larger than that recorded in the bottom layer. The reason for measuring relatively small lateral displacements is related to the type of fine materials (densified fine tailings) used and the relatively low excitation frequency of 1.0. Dynamic amplification is small at low excitation frequency.

### Pore water pressure response and liquefaction analysis

Numerous liquefaction triggering criteria have been proposed and adopted to describe or determine liquefaction of natural or man-made (tailings) soils. These criteria comprise excess pore pressure-based criteria, strain/deformation-based liquefaction criteria, energy-based liquefaction criteria, and strength-based criteria. Each of these methods has advantages, but also limitations, as described in various previous studies (e.g., [91]).

The excess pore pressure-based criteria are adopted in this study. The development and the dissipation of the excess pore water pressure or the pore pressure-based criteria have been extensively used in evaluating the liquefaction potential of natural and man-made (tailings) in the laboratory, particularly in shaking table tests (e.g., [37, 49, 61, 85, 87]). The ratio of the excess pore water pressure (${r}_{u}$) is a key parameter that is used as an indication for the liquefaction occurrence. In other words, the criterion, r_u_, defines the onset of liquefaction. When the ratio of the excess pore water pressure (${r}_{u}$) is equal to 1, the liquefaction occurs [11, 61, 62, 84]. However, it has been also shown that the value of r_u_ at liquefaction triggering is affected by soil densities and loading conditions. Denser soils subjected to larger shear stress ratios generate lower pore pressure ratios r_u_ at the onset of liquefaction [91]. Denser soils have limited shear strain potential due to their strong dilative behavior upon continuous shear deformation [91].

This ratio, ${r}_{u}$, can be calculated via Eq. 1

1 $$r_{u} = \Delta u/\sigma^{\prime}_{vo}$$

In Eq. 1, $\Delta u$ represents the excess pore water pressure generated during shaking loading and ${\sigma {^{\prime}}}_{vo}$ represents the initial vertical effective stress prior to shaking loading.

**Fig. 8:**
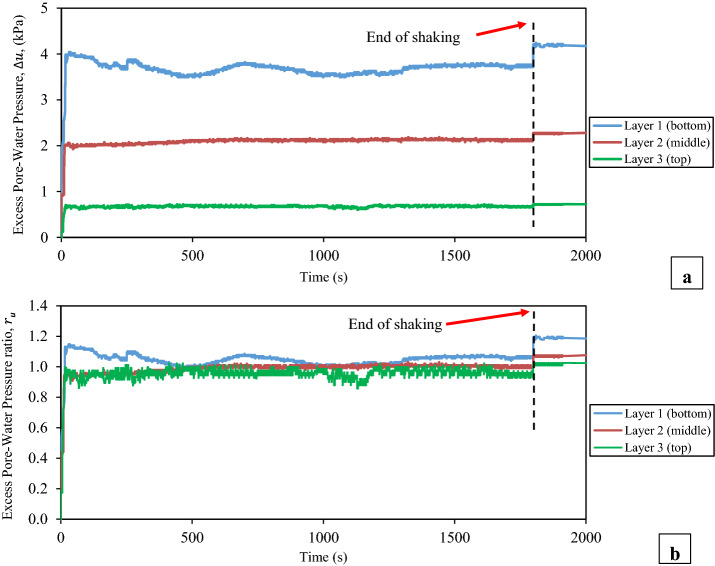
Time histories of the excess pore water pressure at the intermediate depth of each layer of the thickened tailings deposit during shaking loading. **a** Measured excess pore water pressures (∆*u*). **b** Excess pore water pressure ratios (*r*_*u*_)

**Fig. 9:**
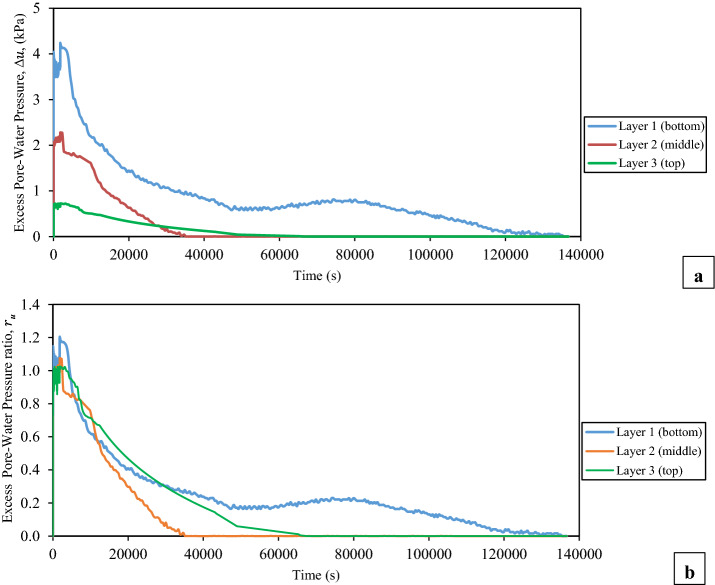
Time histories of the excess pore water pressure at the intermediate depth of each layer of the thickened tailings deposit during and after shaking loading. **a** Measured excess pore water pressures (∆*u*). **b** Excess pore water pressure ratios (*r*_*u*_)

**Fig. 10:**
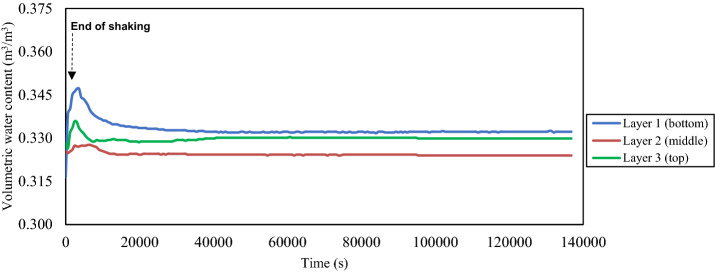
Time histories of volumetric water content at the intermediate depth of each layer of thickened tailings deposit during and after shaking

The measured time histories of the excess pore water pressure at the intermediate depth of each layer of the tested thickened tailings and the corresponding excess pore water pressure ratio are presented in terms of during and after shaking loadings in Figs. 8 and 9, respectively. The excess pore water pressure at the intermediate depth of each layer of the tested tailings started to build-up immediately once the shaking loading was applied, as shown in Fig. 8. This excess pore water pressure was continuous to build-up until reaching a peak value which then it did not alter until the shaking loading was terminated. The contraction response of the tested tailings had caused the built-up of this excess pore water pressure, as illustrated in Figs. 12 and 13, which will be discussed later in the next section ("Vertical displacement/settlement response" section). Volume dilation of the tested tailings, as the results of the applied shaking loading at very low vertical effective vertical stress, had led to the somewhat unchanged in the excess pore water pressure beyond this peak value [61], as evidenced in Figs. 12 and 13. The bottom layer needed the longest time in order to reach the peak value of the excess pore water pressure ($\Delta u$) when compared with the other two layers, as revealed in Fig. 8. Because of water flow and pressure redistribution takes time, such a delay occurred. Therefore, there is often a delay between the time of cyclic shaking and time of attainment the peak excess of pore water pressure or liquefaction failure [54, 75]. Also, it has been observed from this figure that the bottom layer had the highest peak value of the excess pore water pressure ($\Delta u$), which then followed by the middle layer and lastly by the top layer. This means that as the depth increased, the peak value of the excess pore water pressure ($\Delta u$) became larger. Such higher peak value of the excess pore water pressure ($\Delta u$) at the bottom layer might be partly related to the downward flow of pore water that came from the middle layer to the bottom layer as supported by the results of the volumetric water content as revealed in Fig. 10 (later will be discussed). An additional factor that could have contributed to this higher peak value is the fact that the shear strain profile for a column subjected to a vertically propagating horizontal shear wave varies from zero at the surface and increases with depth [73]. This indicated that this pore water pressure in the bottom layer is generated partially by pore pressures generated in the bottom layer and partially by the inflow of pore water from the upper layer. Moreover, from Fig. 10, it is also noticeable that an upward flow of pore water from the lower part of the thickened tailings deposit to the upper (top) layer took place during shaking. In addition, the observations of Naesgaard and Byrne [54] on layered soils supported the previous description as they reported that earthquake shaking of layered soils persuades pore water gradients, the inflow of pore water in some areas or layers and outflow of pore water in some areas. Similar behaviour was reported by Tohumcu Özener et al. [79] who investigated the pore water pressure generation and related liquefaction in layered sand soils under shaking table equipment. Furthermore, the top layer needed longer time to reach the peak value of the excess pore water pressure during shaking in comparison to the middle layer. This can be explained by the fact that water flow and pressure redistribution takes time. Accordingly, there is often a delay between time of shaking and time of reaching the maximum excess of pore water pressure [54, 75]. The ratio of the excess pore water pressure (${r}_{u}$), during the shaking, was noticed to be greater than 0.95 at all depths of the tested tailings, as revealed in Fig. 8.

**Fig. 11:**
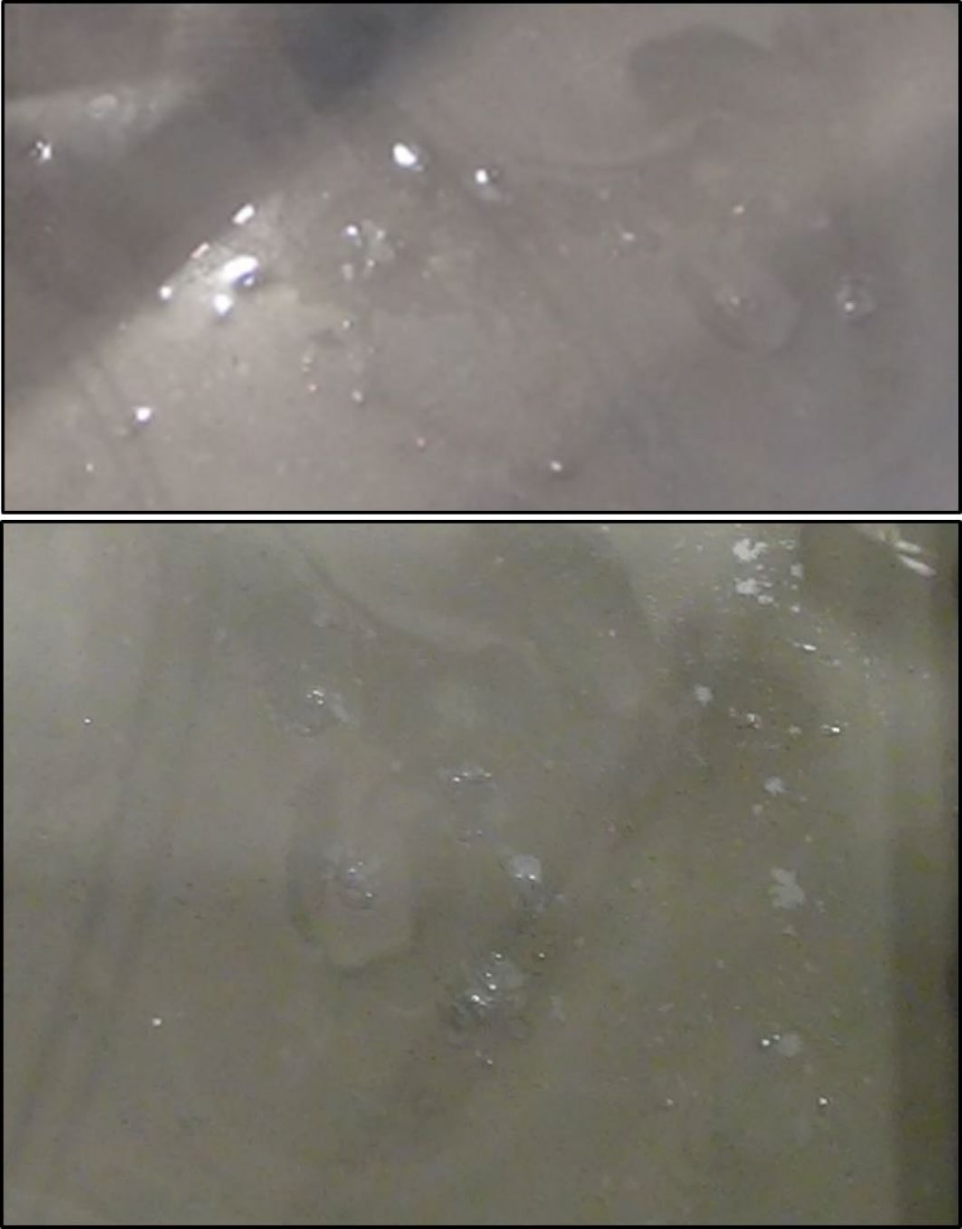
Tailings boils observed on the ground surface of the thickened tailings deposit after liquefaction

The termination of shaking loading can be pointed out by the red arrows, as displayed in Fig. 8. An interesting point in this figure is the sudden building-up in the excess pore water pressure ($\Delta u$) that was observed throughout all depths of the tested thickened tailings as soon as the applied shaking loading was terminated. This led the ratio of the excess pore water pressure (${r}_{u}$) to be built-up slightly to be over 1 in general. Based on the above observations, it can be concluded that the tested thickened tailings are potentially liquefiable material under the used input shaking loading conditions. This finding is in agreement with the finding of Antonaki et al. [4] who have assessed the liquefaction of tailings under the centrifuge testing. It has been seen that as the depth of the tested tailings increased, the amount of this sudden building-up became greater. Such resulted behaviour can be explained by the difference between the three layers in terms of the amount of the vertical total stress where the larger vertical total stress was related to the deeper depth. The termination of the shaking loading is considered to be responsible for the occurrence of such sudden increase, which permitted the volume contraction of partially suspended tailings grains, thus producing further pore water pressure at all depths [61]. The observed responses herein agree with those reported in the literature [61, 62] for hard rock tailings. The process of the upward and downward flow of pore water at the tested thickened tailings deposit could be considered as a further reason that might lead to this sudden increase of excess pore water pressure in the top layer (Layer 3) and bottom layer (layer 1). The pore-water flow upward from the lower part of the thickened tailings deposit to the upper (top) layer while it flows downward from the lower part of the middle layer of the thickened tailings deposit to the bottom layer (Layer 1). This downward water flow is enabled by the presence of drainage layer at the bottom of the tailings deposit (Fig. 4). This previously explained process is supported through both the evolution of the volumetric water content (VWC), which was observed to be increased in the intermediate of the top and bottom layers during the applied shaking and up to about 40 min after the end of applied shaking as revealed in Fig. 10, and with the appearance of tailings boils at the ground surface of the top layer of the thickened tailings deposit (Fig. 11). Such manifestation was observed following the liquefaction of Mailiao silty sand [82] and following the liquefaction of thickened fine mine tailings [4]. This manifestation was attributed to the expel of water together with the grains to the ground surface of the tested tailings. Moreover, Fig. 10 shows the increase in VWC observed in the bottom layer is higher than that in the top layer (it should be underlined that the VWC was only measured at the middle of each layer, not over the entire height of each layer). This downward flow of more water to the bottom layer might have been facilitated by desiccation-induced microcracks that appeared on the surface of the first layer of thickened tailings after 3 days of drying, i.e. before the deposition of the second layer. It is well acknowledged that cracks increase the permeability of soil or tailings deposits [70, 71].

The value of this sudden increase in excess pore water pressure, at all depths of deposit, stayed stable for a short period of time after the shaking loading was terminated, as revealed in Fig. 9. Then, the generated pore water pressure started the process of its dissipation, which took a long period of time (approximately 35,000–130,000 s). Pépin et al. [61, 62] tested the tailings under cyclic loading conditions and found that the tailings needed a long period of time in order to reach the full dissipation of its excess pore water pressure. However, several studied (e.g. [84]) have indicated that the required time for dissipation of the excess pore water pressure of sandy soil was short. The difference between the tested tailings and the sandy soil, in the period of dissipation, can be due to the relatively lower hydraulic conductivity (~ 10^–5^—10^–7^ m/s) that associated with the uncracked thickened tailings [9, 67] than that in sandy soil (~ 10^–3^—10^–5^ m/s). An important observation was noticed in Fig. 9, where the full dissipation of the excess pore water pressure at the bottom layer needed the greatest period of time when compared with the other two layers. Such observation can be associated with the inflow of pore water as the largest evolution of volumetric water content (VWC) at the intermediate depth of the bottom layer was noticed after the termination of the applied shaking loading, as revealed in Fig. 10. The excess pore water pressure at the top layer dissipated slower than that at the middle layer. The reason for that might be related to the transition of water from the middle layer to the top layer which is considered in consistence with the lower evolution of the volumetric water content at the middle layer than at the top layer, as revealed in Fig. 10, and also with the observations of other researchers [54, 75] who investigated the seismic liquefaction of layered soils. Figure 10 also shows that the initial values of VWC for each layer are slightly lower than the VWC values at the end of the test. This could be explained by the rearrangement of the tailings particles located around the 5TE sensor to a denser structure.

### Vertical displacement/settlement response

**Fig. 12:**
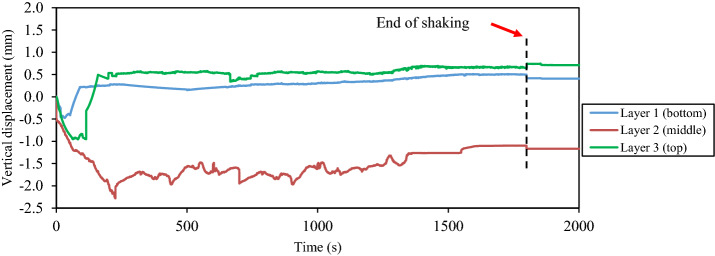
Time histories of the vertical displacement at the intermediate depth of each layer of the thickened tailings deposit during shaking loading

**Fig. 13:**
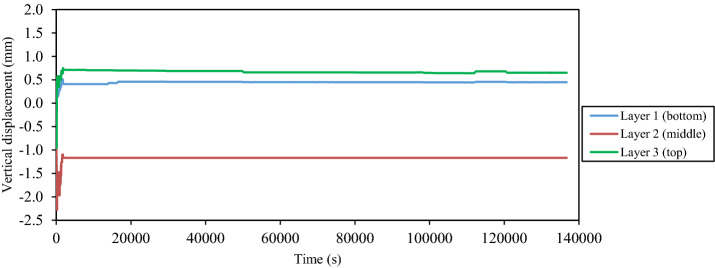
Time histories of the vertical displacement at the intermediate depth of each layer of the thickened tailings deposit during and after shaking loading

During and after the shaking loadings, the measured time histories of the vertical displacement at the intermediate depth of each layer of the tested thickened tailings are presented in Figs. 12 and 13, respectively. The red arrow in Fig. 12 depicts the termination of the shaking loading. Two types of responses that the tested tailings passed through during the shaking loading, namely, the volume contraction and dilation, as deduced from Fig. 12. This figure revealed that all layers of tested tailings were experienced settlement initially (i.e., volume contraction) as soon as the shaking loading was applied. Generally, the value of this settlement was approximately 0.5 mm for the bottom layer, 2.3 mm for the middle layer, and 1.0 mm for the top layer, respectively. This indicates that the tailings deposit at the bottom layer had the lowest settlement as compared with the other two layers. The reason for such a response could be due to the difference in terms of the duration of the drying (desiccation) between these layers as the top and middle layers were experienced a lower duration of drying (desiccation) than that at the bottom layer. The previous investigation conducted by Daliri [16] revealed that the response of tailings changed from contraction to dilation when they subjected to somewhat degree of drying or desiccation. Following the volume contraction (i.e., settlement response), the tested deposit transformed to the other response (i.e., volume dilation) which as a result of the uplift of the plastic plates at all layers. The dilation of tested tailings was firstly somewhat developed at the bottom layer then proceeded at the top layer and eventually at the middle layer. The inflow of pore water from the upper portion (middle layer, upper portion of the bottom layer) of tested thickened tailings is considered an additional reason that leads to a lower volume contraction and dilation at the bottom layer. The observation from this current study is considered consistent with that of layered soils as reported in the literature [54, 75]. Following the termination of the shaking loading, it seemed that no substantial difference in the response of the thickened tailings deposit, as revealed in Fig. 13.

## Conclusions

In this research work, a shaking table test was conducted on three thin layers of thickened tailings contained within a flexible laminar shear box. This flexible laminar shear box was subjected to a peak horizontal acceleration of 0.13 g and a frequency of 1 Hz. Based on these considered testing conditions, the following conclusions can be drawn.

Typically, the results from the measured accelerations indicated that the applied input acceleration propagated completely to the bottom and middle layers except for the top layer, which could be due to its strength and stiffness reduction during shaking. Similar measured horizontal displacements were observed at all depths of the layered thickened tailings.

The flow of water and pressure redistribution in the tested layered of thickened tailings were occurred due to the applied shaking loading. Both the bottom and top layers were subjected to the inflow of water, while the middle layer is influenced by the outflow of water. The behaviour of the tested layered thickened tailings in terms of excess pore water pressure, liquefaction, and vertical displacement significantly influenced by such outflow and inflow of water.

The excess pore water pressure at the intermediate depth of each layer of the thickened tailings deposit was seen to build-up immediately and continuously until attainment a peak value during the applied shaking. Such a built-up was related to the volume contraction of this type of tailings. Additionally, the inflow of pore water into both the bottom and top layers played a role in such a built-up of excess pore water pressure. Once the applied shaking was terminated, there was a sudden building-up in the excess pore water pressure of the layered thickened tailings. Typically, this caused the ratio of the excess pore water pressure to be slightly larger than unity which revealed that the layered thickened tailings are susceptible to liquefaction under the considered testing conditions. This sudden building-up in the excess pore water pressure is occurred as a result of two factors, namely, (i) the volume contraction of partially suspended tailings grains which producing further pore water pressure; (ii) the movement of upward pore water to the top layer and downward pore water to the bottom layer which leads to produce further pore water pressure. The full dissipation of the excess pore water pressure of the tested layered thickened tailings was seen to be occurred after a long period of time as expected due to the low permeability of such tailings.

During the shaking loading, the layered thickened tailings were observed to be subject to two types of responses, namely, the volume contraction and dilation as per the measurements of the vertical displacements. Since the bottom layer of the deposit was subjected to higher duration of drying (desiccation) than that at the top and middle layers and the more inflow of pore water into the bottom layer of the deposit, the bottom layer of the deposit was experienced less settlement (i.e., volume contraction) than that at the top and middle layers. There was an insignificant difference in the vertical displacements of the layered thickened tailings after the termination of the shaking loading.

Based on the findings mentioned above, alternative disposal methods such as paste tailings (tailings densified to a solid content between 70 to 85%) and dry stack tailings (filtered tailings, tailings densified to a solid content higher than 85%) might be considered to increase the resistance to cyclic liquefaction, specifically in regions located in high seismic activities. The obtained results in this research work provide knowledge about the seismic behaviour and liquefaction potential thickened tailings deposit, under the seismic loading conditions applied in this study, specifically for practitioners and researchers involved in the mining industry, and engineering geological or geotechnical research community.
